# Supplementary material for: U-to-C RNA editing by synthetic PPR-DYW proteins in bacteria and human culture cells
Source: Commun Biol. 2022 Sep 15;5:968. doi: 10.1038/s42003-022-03927-3 (PMC9478123; doi:10.1038/s42003-022-03927-3)
Supplement: Supplementary file 2 — Supplementary information [file 42003_2022_3927_MOESM2_ESM.pdf]

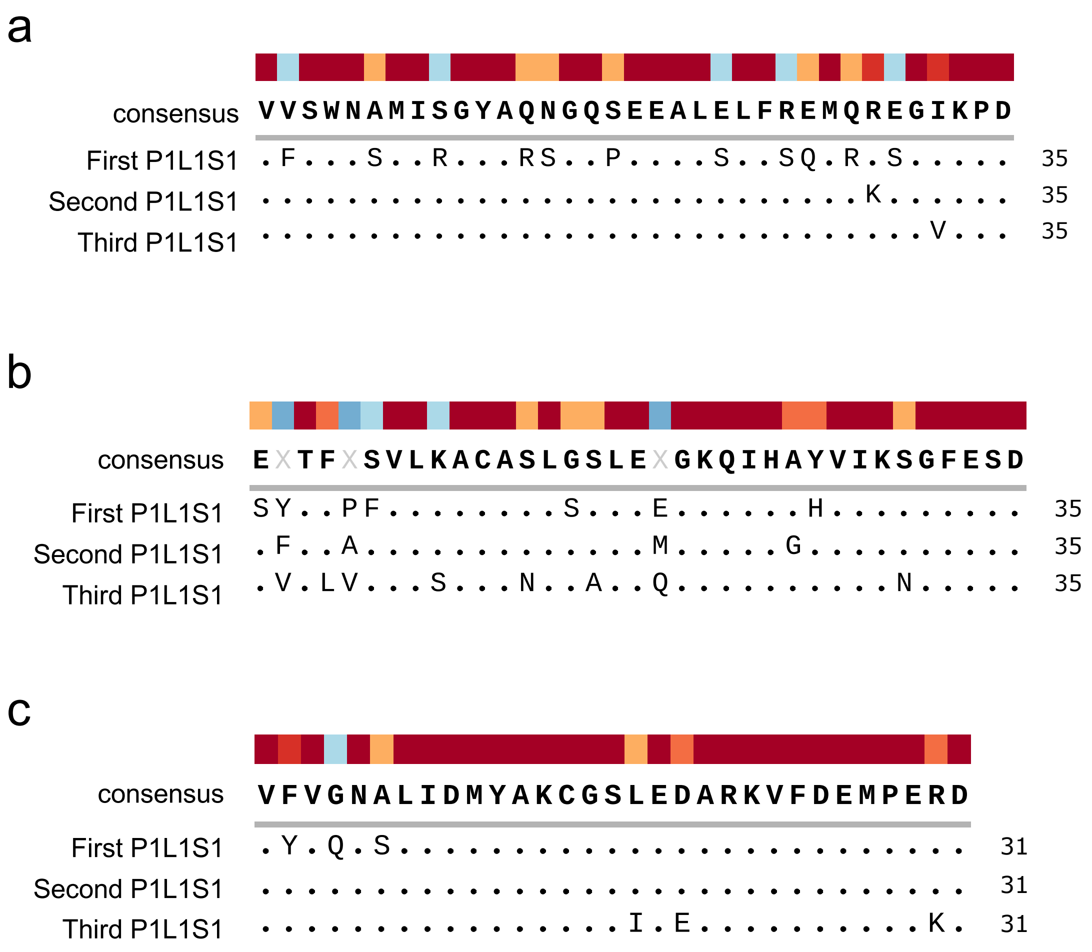

Supplementary Figure 1. Amino acid sequence comparison of three P1L1S1 triplets of PPR motifs. The P1 (a), L1 (b) and S1 (c) motifs of the first, second and third triplets composing the PLS array are aligned. Consensus sequence is shown on the top. The amino acids matching the consensus sequence are marked with a dot. Higher conservation is indicated by warm colors (such as brown and red) and lower conservation by cool colors (such as blue).

a

```
consensus  VVTWNAMIXGYGXXXEGXMAXQCFEDMQQQGVKPS
KP1        ......LLN..AQHND.Q..I....E......E..   35
KP2        ........N...QSN..HK.IE..QE.L.D..Q..   35
KP3        ......L.S...TCD..HK.LA...E.LVA.IQ..   35
KP4        ..S.....A..AQCGNIEL.R........E.I...   35
KP5        ..S.....R...MNH..N..V.......K..I...   35
KP6        ..A..S..K...MNH..K..V.......K......   35
KP7        ........N...MNHD.Q..I..............   35
```

b

```
consensus  XVTFTCLLXACSHAGLVXEGQQYFKMMXEXHGIXPR
KP1        DA......V....E...N.........V.D...V..   36
KP2        ET......V....E...N..R....A.V.E...M..   36
KP3        DA......V....E...H..RKH.DS.A.Q...K..   36
KP4        E....SI.A........D..CK...S.K.D...I..   36
KP5        A.......T...R.N..VK........RGKY..T..   36
KP6        A.......T.....S..S...E.....R.EY..A..   36
KP7        S.......T........N.........R.DY..V..   36
```

c

```
consensus  VXHYNCMVDLLGRSGHLDEAEKMLXTXXXXXX
KP1        .E...........A.Y........LSF--ESN   30
KP2        IE.....I.....A..........LSCEGQSN   32
KP3        TH...........A.Y.N...NL.Q.I--QPD   30
KP4        .Y...................N..Q.MPFEND   32
KP5        .H...................N..Q.MPFEND   32
KP6        .D..T......A.....H....L.E.APCPPS   32
KP7        .E..T......A.....Y....F.EML-CPPS   31
```

d

```
consensus  XVGWGSLLSACKTHGDVERGXRCFXQLVXLDPES
KP1        V...........S.......R...DH..K.E...   34
KP2        D..F....N..RS...I...T...ES..R...Q.   34
KP3        V..L....N...S...I..AMW..ESI.R...Q.   34
KP4        II......T..R.Y.N..L.R...D.I.Q...H.   34
KP5        KET..A.......Y.E..L.L...Q...QMN...   34
KP6        EGT..A.......Y.E..L.L...Q...Q.N...   34
KP7        E...................R...E...K.....   34
```

e

```
consensus  ASXYVLMANIYAXAGRWXDADRIEELRKHAGAXK
KP1        ..A...LG....N....K.V....S...S...W.   34
KP2        ..A.........D....T.V....R...S...S.   34
KP3        ..A......A..D....T.V....TQ.MV...N.   34
KP4        ..G....SG..SD.QM.E..AK.....Q....W.   34
KP5        .AS.........N....D..NK..........K.   34
KP6        .AW.....D...G....D..Y...........K.   34
KP7        .AW.........N....D..............K.   34
```

Supplementary Figure 2. Amino acid sequence comparison of seven designer KP proteins. The amino acid sequences of P2 (a), L2 (b), S2 (c), E1 (d) and E2 (e) motifs are aligned and compared to a consensus sequence shown on the top. The amino acids matching the consensus sequence are marked with a dot. Higher conservation is indicated by warm colors (such as brown and red) and lower conservation by cool colors (such as blue).

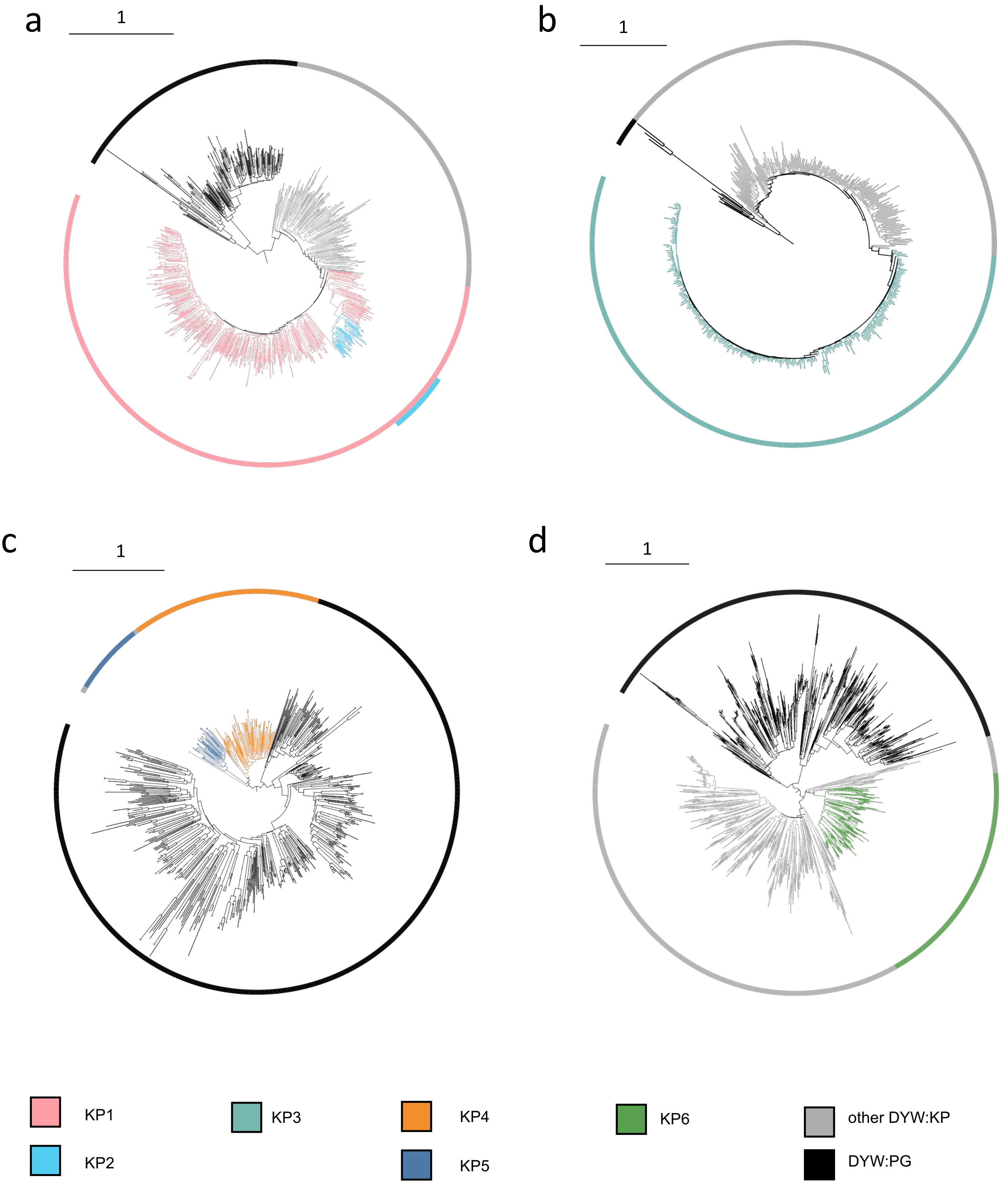

Supplementary Figure 3. DYW:KP subclades used in the design of the U-to-C editing factors. Apporximately maximum-likelihood phylogenetic trees on DYW C-terminal domains isolated in hornwort (a), lycophyte (c) and fern (d) transcriptomes and *Anthoceros angustus* genome (b), rooted using DYW:PG as an outgroup. Colour code indicates the subgroups of DYW:KP proteins isolated to design the DYW:KP proteins. Visualisation was with iTOL (Letunic and Bork 2016).

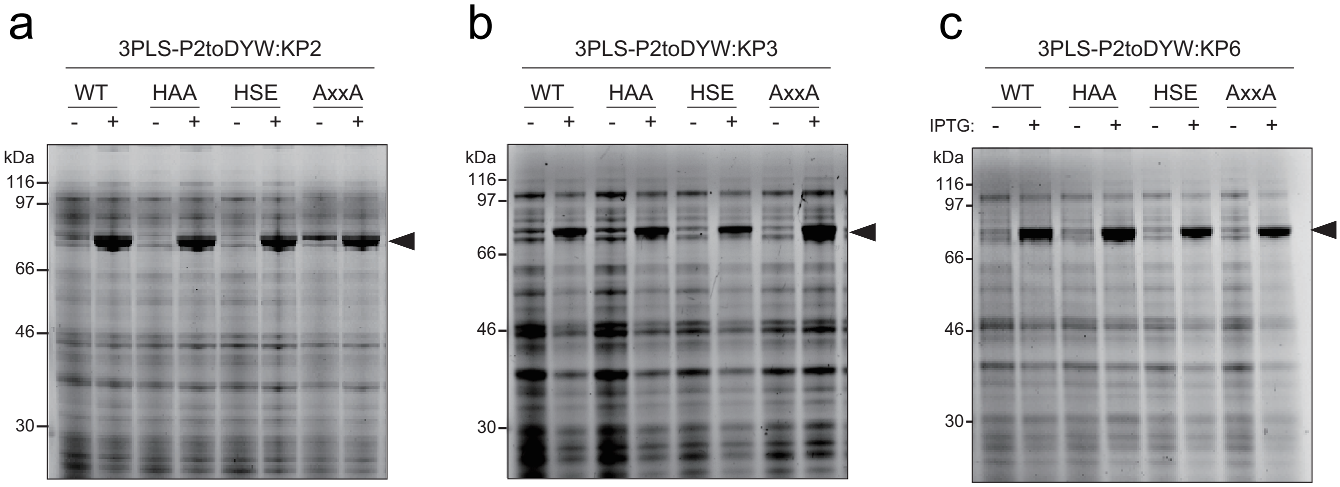

Supplementary Figure 4. The expression of DYW:KP catalytic mutant proteins. KP2 (a), KP3 (b) and KP6 (c) protein expression was verified by SDS-PAGE after loading bacterial lysates before (-) and after (+) 18 h IPTG induction.

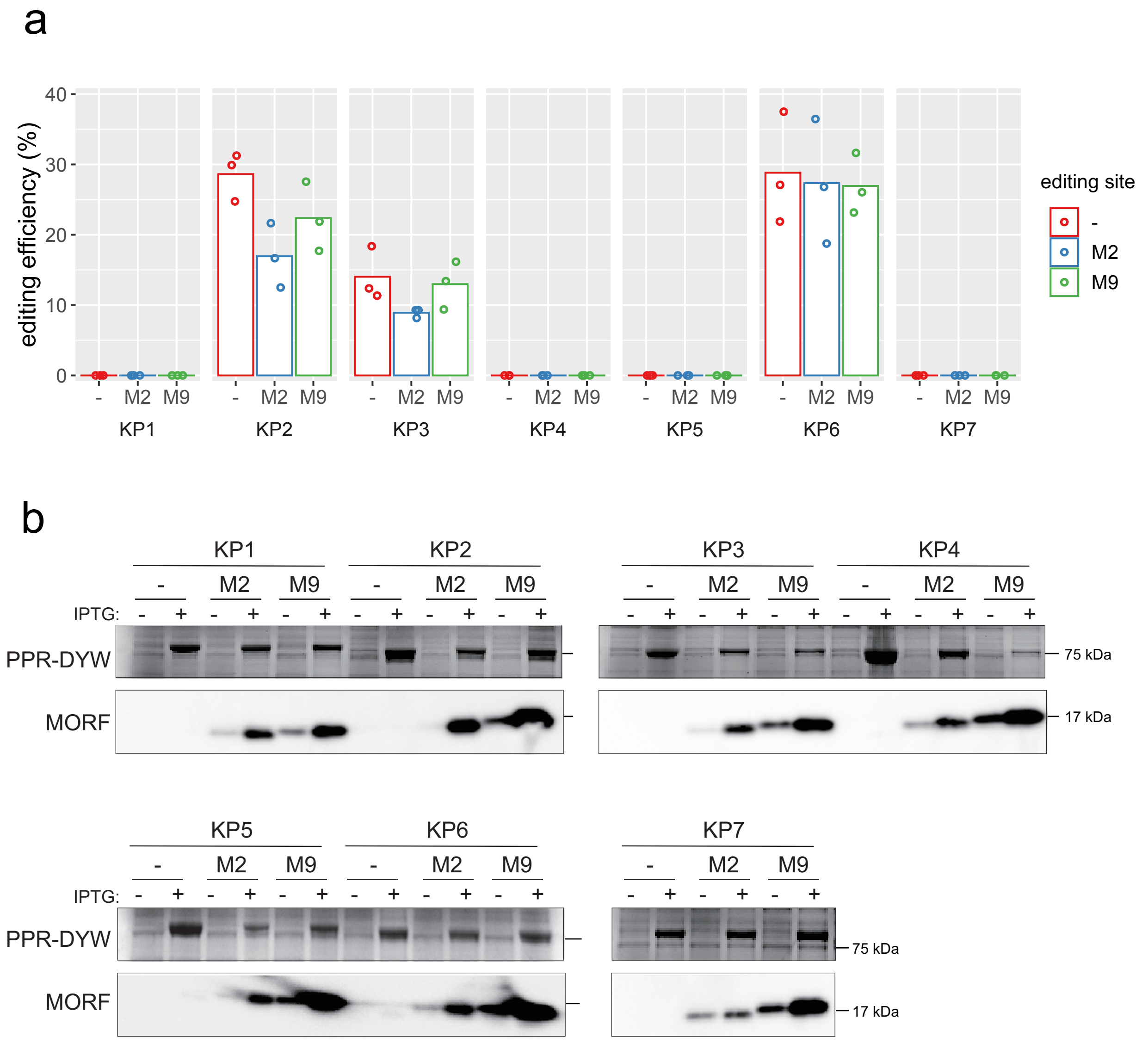

Supplementary Figure 5. U-to-C editing activity with MORF proteins. (a) Editing efficiency of DYW:KP proteins in the absence (-) or presence of MORF2 (M2) or MORF9 (M9). The bar height corresponds to the mean of editing efficiency between three biologically independent replicates (dots). (b) The expression of the DYW:KP (upper panel) and MORF proteins (lower panel) were verified by SDS-PAGE or western blotting (1:10,000 Anti-His-tag mAb-HRP-DirectT, MBL D291-7) after loading bacterial lysates before (-) and after (+) 18 h IPTG induction.

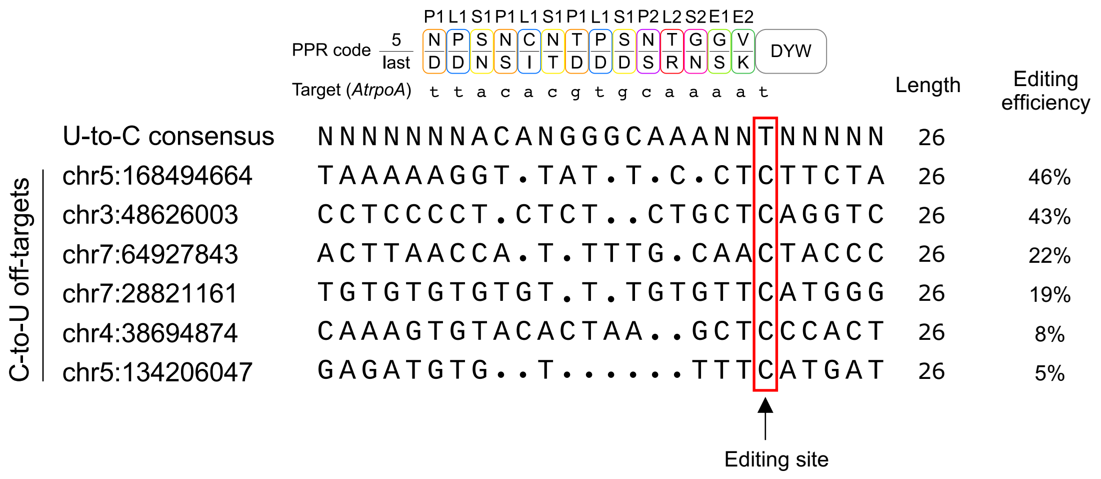

Supplementary Figure 6. Nucleotide sequence comparison of six C-to-U off-target editing sites with the U-to-C off-target site consensus sequence. The consensus was derived from EMBOSS:cons. The nucleotides matching the consensus sequence are marked with a dot.

a

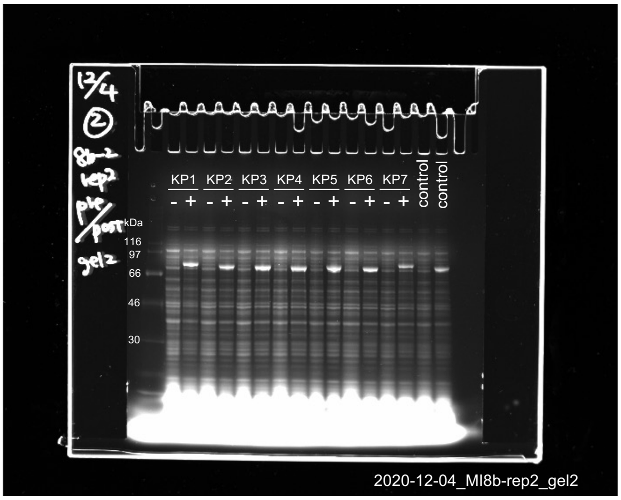

b

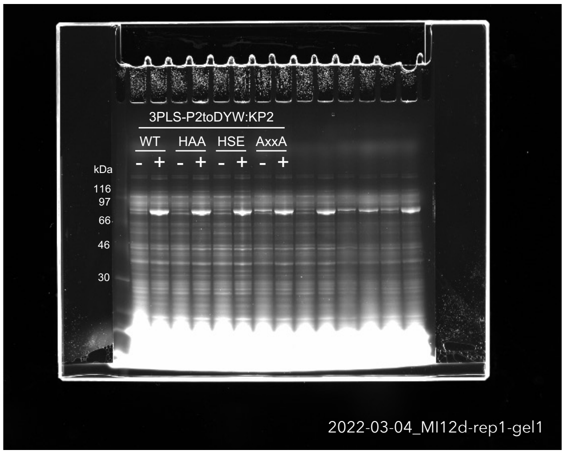

c

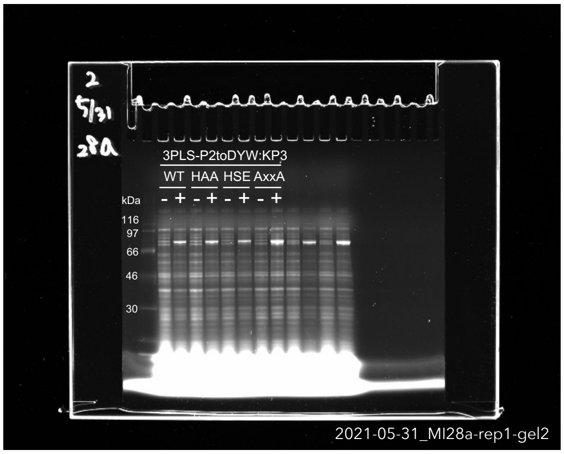

d

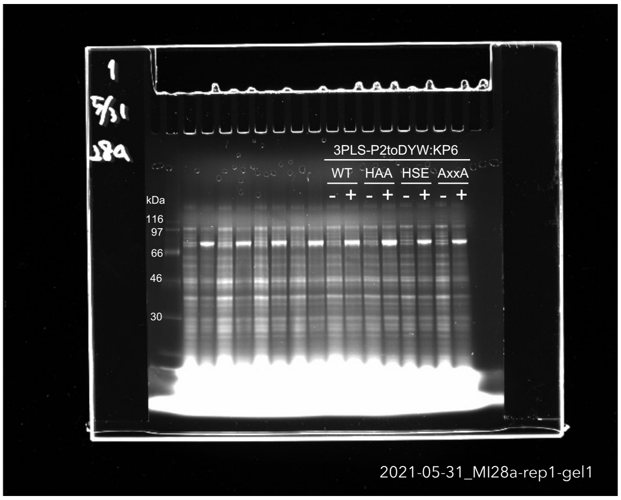

Supplementary Figure 7. Uncropped and unedited images used in Figure 3 and Supplementary Figure 4
SDS-PAGE gels shown in Figure 3b (a), Figure S4a (b), Figure S4b (c) and Figure S4c (d).

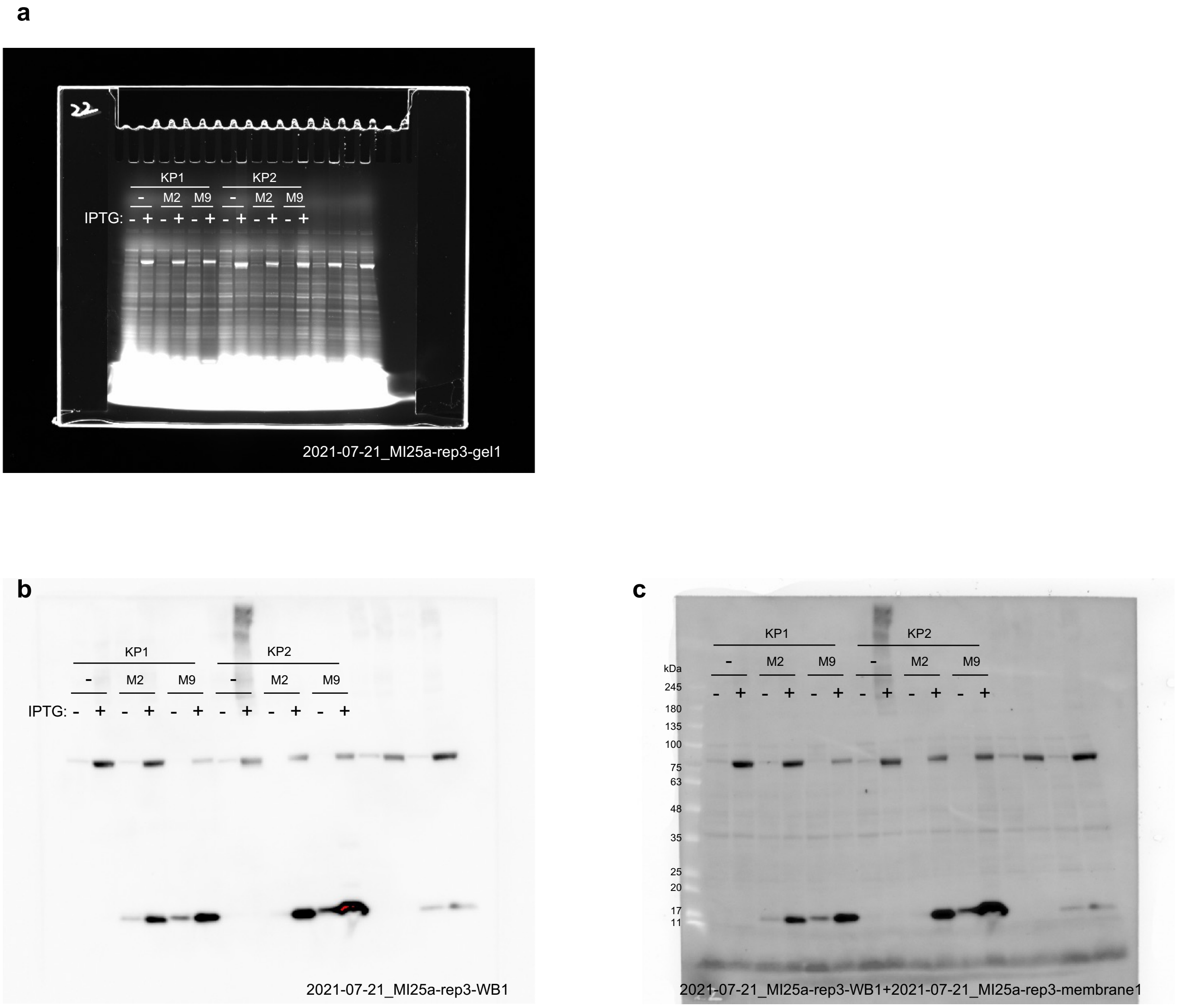

Supplementary Figure 8. Uncropped and unedited images corresponding to the SDS-PAGE gel and western blot images shown in Supplementary Figure 5 for KP1 and 2.
(a) SDS-PAGE gel, (b) blot with His-tag and (c) merged with size marker showing the expression of KP1, 2 and MORF proteins.

a

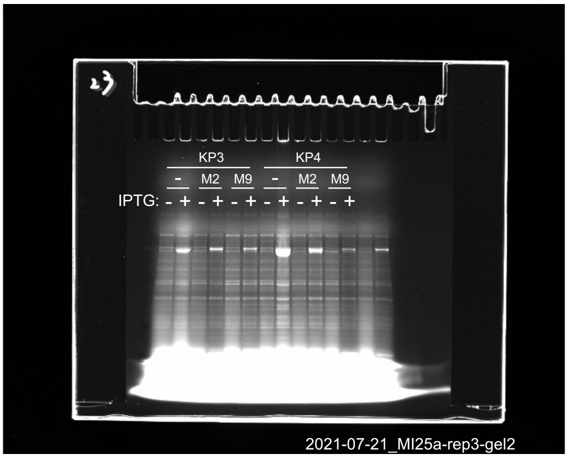

b

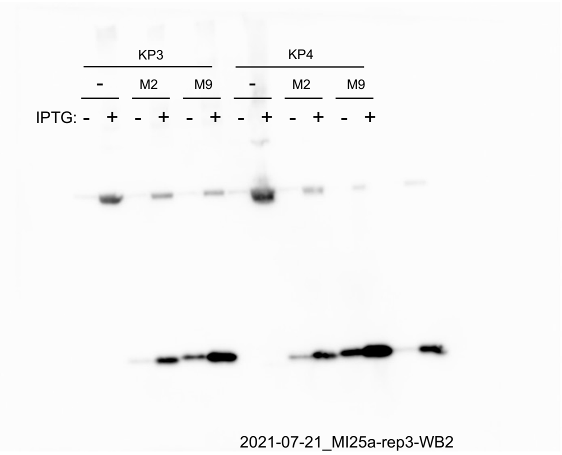

c

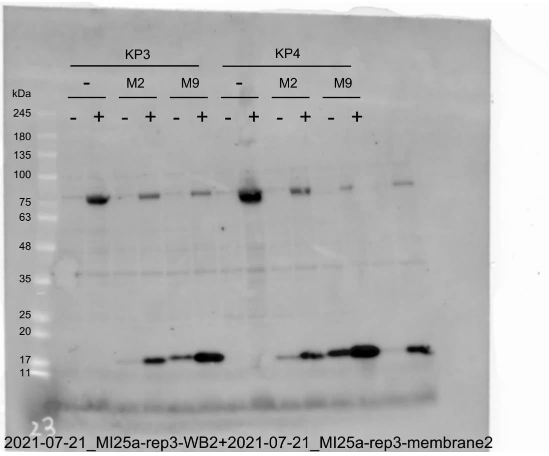

Supplementary Figure 9. Uncropped and unedited images corresponding to the SDS-PAGE gel and western blot images shown in Supplementary Figure 5 for KP3 and 4.
(a) SDS-PAGE gel, (b) blot with His-tag and (c) merged with size marker showing the expression of KP3, 4 and MORF proteins.

a

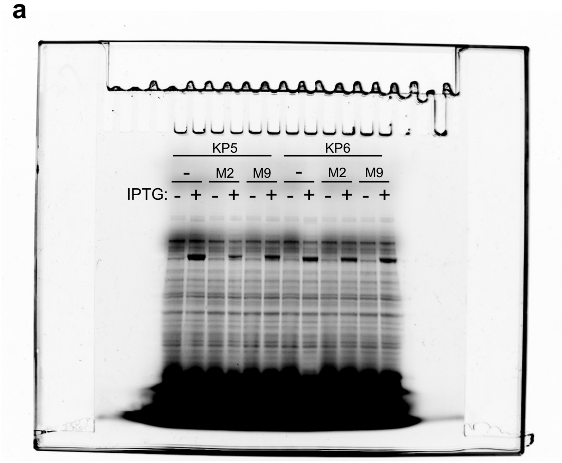

2021-08-04_MI25a-rep3-gel3

b

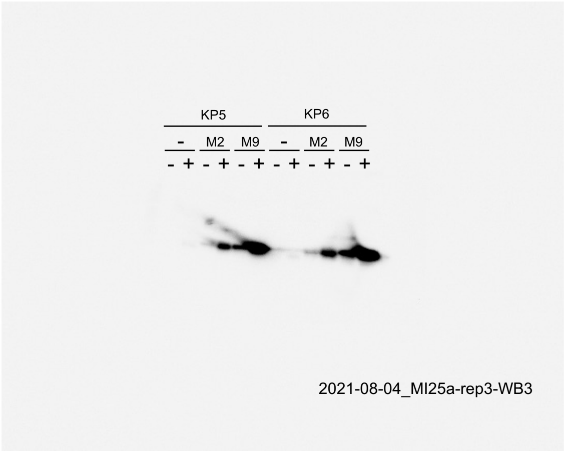

c

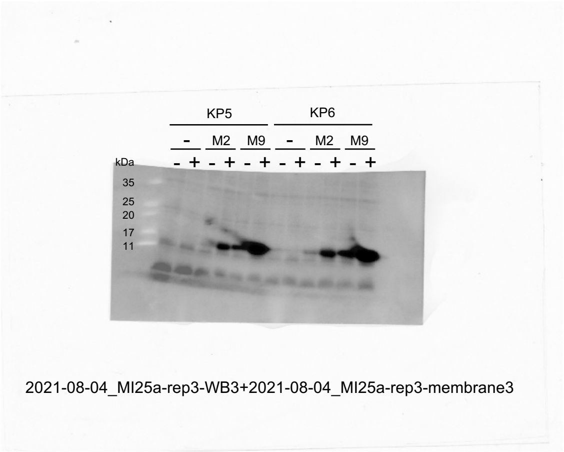

Supplementary Figure 10. Uncropped and unedited images corresponding to the SDS-PAGE gel and western blot images shown in Supplementary Figure 5 for KP5 and 6.
(a) SDS-PAGE gel, (b) blot with His-tag and (c) merged with size marker showing the expression of KP5, 6 and MORF proteins.

a

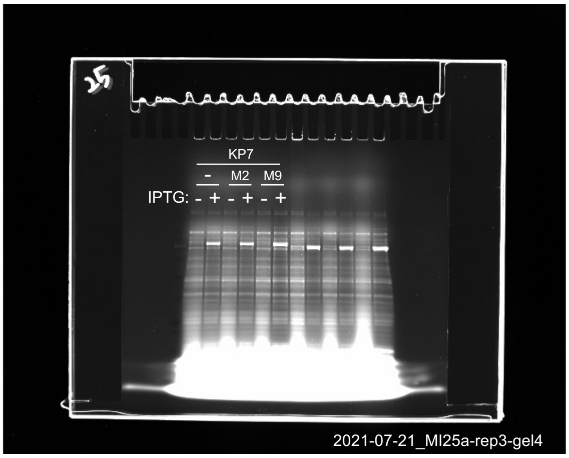

b

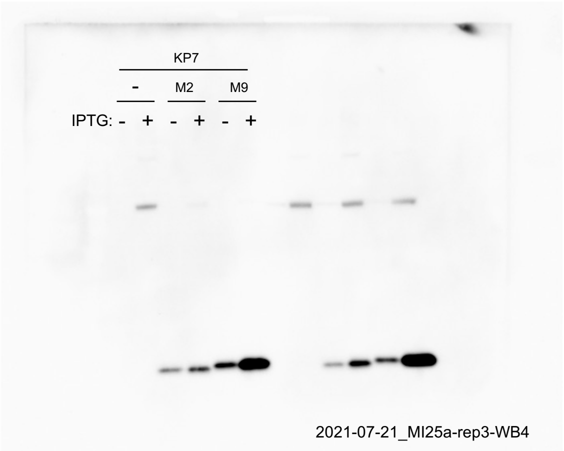

c

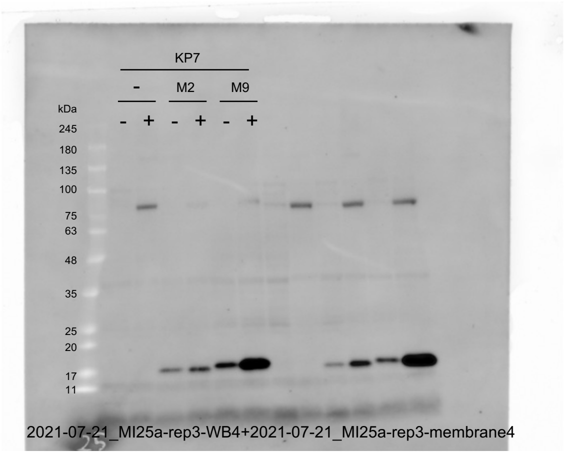

Supplementary Figure 11. Uncropped and unedited images corresponding to the SDS-PAGE gel and western blot images shown in Supplementary Figure 5 for KP7.
(a) SDS-PAGE gel, (b) blot with His-tag and (c) merged with size marker showing the expression of KP7 and MORF proteins.

Supplementary Table 1. The number of sequences used to design the PLS domain

	P1	L1	S1
First	3,363	3,741	3,845
Middle	18,643	22,364	28,337
Last	12,882	13,871	15,593

Supplementary Table 2. The number of sequences used to design the KP domains

	P2	L2	S2	E1	E2	DYW
KP1	314	315	342	432	476	301
KP2	20	20	22	36	40	37
KP3	209	219	221	225	229	216
KP4	44	57	48	67	73	66
KP5	22	19	24	24	28	26
KP6	221	243	219	276	301	287
KP7	411	432	282	540	580	658

Supplementary Table 3. Oligonucleotides used in this study

Name	Sequence (5' -> 3')	Purpose
Trx-Esp3I-F	GCATCGTCTCATGGCCGGAGGGAGCGATAAAATTATTCACCTGACT	clone Trx-6His-TEV in modified pET21b vector and mammalian expression vector
TEV-Esp3I-R	TCGTCTCTACTAGAAGACATGTGACAACAGAATTACCCATGGCGC	clone Trx-6His-TEV in modified pET21b vector
Esp3I-BpiI-KP1-F	TCGTCTCATAGTGAAGACATGACGTTGTAACCTGGAATGCCTT	clone KP1 in modified pET21b vector
Esp3I-BpiI-KP2-F	TCGTCTCATAGTGAAGACATGACGTGGTGACTTGGAACGCTAT	clone KP2 in modified pET21b vector
Esp3I-BpiI-KP3-F	TCGTCTCATAGTGAAGACATGACGTCGTTACTTGGAACGCCCT	clone KP3 in modified pET21b vector
Esp3I-BpiI-KP4-F	TCGTCTCATAGTGAAGACATGACGTTGTCTCTTGGAATGCAAT	clone KP4 in modified pET21b vector
Esp3I-BpiI-KP5-F	TCGTCTCATAGTGAAGACATGACGTCGTCAGCTGGAATGCGAT	clone KP5 in modified pET21b vector
Esp3I-BpiI-KP6-F	TCGTCTCATAGTGAAGACATGACGTTGTGGCTTGGAACTCAATGAT	clone KP6 in modified pET21b vector
Esp3I-BpiI-KP7-F	TCGTCTCATAGTGAAGACATGACGTTGTTACGTGGAACGCGAT	clone KP7 in modified pET21b vector
Esp3I-KP1-R	TCGTCTCTGTTAGTGTCGGTCCCCACAACTAC	clone KP1 in modified pET21b vector
Esp3I-KP2-R	TCGTCTCTGTTATGGTCGTCCTCCGCAACTGC	clone KP2 in modified pET21b vector
Esp3I-KP3-R	TCGTCTCTGTTATGGCCGTCCCTTGCATGAGC	clone KP3 in modified pET21b vector
Esp3I-KP4-R	TCGTCTCTGTTAGTAGTAGTCTCCGCAACTGC	clone KP4 in modified pET21b vector
Esp3I-KP5-R	TCGTCTCTGTTACCACATATCGCCGCATGAGC	clone KP5 in modified pET21b vector
Esp3I-KP6-R	TCGTCTCTGTTAGAACATGTCTCCGCAGGAGC	clone KP6 in modified pET21b vector
Esp3I-KP7-R	TCGTCTCTGTTAGTAGCGATCTCCGCAACTGC	clone KP7 in modified pET21b vector
BpiI-PLS-F	TGAAGACATTCACATGGAATTCCATGATTAGGGGC	clone PLS in modified pET21b vector
BpiI-PLS-R	TGAAGACATCGTCTTTCTCGGGCATTTCATC	clone PLS in modified pET21b vector
rpoA-C-Esp3I-R	GCATCGTCTCTTAGCTCTCAGATTTTGCACGTGTAAT	clone Trx-6His-TEV-3PLS-DYW-rpoA in mammalian expression vector
rpoA-U-Esp3I-R	GCATCGTCTCTTAGCTCTCAAATTTTGCACGTGTAAT	clone Trx-6His-TEV-3PLS-DYW-rpoA in mammalian expression vector
KP3-HAA-mutF	CATGCAGCGAAGCTGGCACTGGCATTC	mutate active site in KP3
KP3-HAA-mutR	CAGCTTCGCTGCATGTCCACAGAGCG	mutate active site in KP3
KP3-HSE-mutF	TGGACATTCAGAGAAGCTGGCACTGG	mutate active site in KP3
KP3-HSE-mutR	TTCTCTGAATGTCCACAGAGCGCGTC	mutate active site in KP3
KP3-AxxA-mutF	TGGCCTCCGACGCCCACTCCTCAACAGAGATTA	mutate active site in KP3
KP3-AxxA-mutR	GGGCGTCGGAGGCCATTCTCAAGTTCTTGGTA	mutate active site in KP3
KP6-HAA-F	CACGCAGCGAAGTTGGCAATTGCATT	modify the nucleotide in *AtrpoA*
KP6-HAA-R	CAACTTCGCTGCGTGCTCACACAGCG	modify the nucleotide in *AtrpoA*
KP6-HSE-mutF	TGAGCACTCAGAGAAGTTGGCAATTG	modify the nucleotide in *AtrpoA*
KP6-HSE-mutR	TTCTCTGAGTGCTCACACAGCGCTGC	modify the nucleotide in *AtrpoA*
KP6-AxxA-mutF	TGGCCAATGACGCCCACAATGCGTCAAAGAT	modify the nucleotide in *AtrpoA*
KP6-AxxA-mutR	GGGCGTCATTGGCCATTCTCAGGTTCTTTGT	modify the nucleotide in *AtrpoA*
rpoA-5C-mutF	CATGTATTACACGTGCCAAATTTGAGAGCTAGCAG	modify the nucleotide in *AtrpoA*
rpoA-5G-mutF	CATGTATTACACGTGCGAAATTTGAGAGCTAGCAG	modify the nucleotide in *AtrpoA*
rpoA-5T-mutF	CATGTATTACACGTGCTAAATTTGAGAGCTAGCAG	modify the nucleotide in *AtrpoA*
rpoA-4C-mutF	CATGTATTACACGTGCACAATTTGAGAGCTAGCAGC	modify the nucleotide in *AtrpoA*
rpoA-4G-mutF	CATGTATTACACGTGCAGAATTTGAGAGCTAGCAGC	modify the nucleotide in *AtrpoA*
rpoA-4T-mutF	CATGTATTACACGTGCATAATTTGAGAGCTAGCAGC	modify the nucleotide in *AtrpoA*
rpoA-3C-mutF	CATGTATTACACGTGCAACATTTGAGAGCTAGCAGCG	modify the nucleotide in *AtrpoA*
rpoA-3G-mutF	CATGTATTACACGTGCAAGATTTGAGAGCTAGCAGCG	modify the nucleotide in *AtrpoA*
rpoA-3T-mutF	CATGTATTACACGTGCAATATTTGAGAGCTAGCAGCG	modify the nucleotide in *AtrpoA*
rpoA-2C-mutF	CATGTATTACACGTGCAAACTTTGAGAGCTAGCAGCGA	modify the nucleotide in *AtrpoA*
rpoA-2G-mutF	CATGTATTACACGTGCAAAGTTTGAGAGCTAGCAGCGA	modify the nucleotide in *AtrpoA*
rpoA-2T-mutF	CATGTATTACACGTGCAAATTTTGAGAGCTAGCAGCGA	modify the nucleotide in *AtrpoA*

Name	Sequence (5' -> 3')	Purpose
rpoA-1A-mutF	CATGTATTACACGTGCAAAAATTGAGAGCTAGCAGCGAG	modify the nucleotide in *AtrpoA*
rpoA-1C-mutF	CATGTATTACACGTGCAAAACTTGAGAGCTAGCAGCGAG	modify the nucleotide in *AtrpoA*
rpoA-1G-mutF	CATGTATTACACGTGCAAAAGTTGAGAGCTAGCAGCGAG	modify the nucleotide in *AtrpoA*
rpoA+1A-mutF	CATGTATTACACGTGCAAAATTAGAGAGCTAGCAGCGAGC	modify the nucleotide in *AtrpoA*
rpoA+1C-mutF	CATGTATTACACGTGCAAAATTCGAGAGCTAGCAGCGAGC	modify the nucleotide in *AtrpoA*
rpoA+1G-mutF	CATGTATTACACGTGCAAAATTGGAGAGCTAGCAGCGAGC	modify the nucleotide in *AtrpoA*
rpoA+2A-mutF	CATGTATTACACGTGCAAAATTTAAGAGCTAGCAGCGAGC	modify the nucleotide in *AtrpoA*
rpoA+2C-mutF	CATGTATTACACGTGCAAAATTTCAGAGCTAGCAGCGAGC	modify the nucleotide in *AtrpoA*
rpoA+2T-mutF	CATGTATTACACGTGCAAAATTTTAGAGCTAGCAGCGAGC	modify the nucleotide in *AtrpoA*
rpoA-BS-Rv	CACGTGTAATACATGTTCCTTCTATTTCT	modify the nucleotide in *AtrpoA*
rpoA-mut-R	TTTGCACGTGTAATACATGTTCCTTCTATT	modify the nucleotide in *AtrpoA*
rpoA-CuCC-F	TATTACACGTGCAAAACTCCAGAGCTAGCAGCGAG	modify the nucleotide in *AtrpoA*
rpoA-CuCA-F	TATTACACGTGCAAAACTCAAGAGCTAGCAGCGAG	modify the nucleotide in *AtrpoA*
rpoA-CuAC-F	TATTACACGTGCAAAACTACAGAGCTAGCAGCGAG	modify the nucleotide in *AtrpoA*
rpoA-CuAA-F	TATTACACGTGCAAAACTAAAGAGCTAGCAGCGAG	modify the nucleotide in *AtrpoA*
rpoA-CcCC-F	TATTACACGTGCAAAACCCCAGAGCTAGCAGCGAG	modify the nucleotide in *AtrpoA*
rpoA-CcCA-F	TATTACACGTGCAAAACCCAAGAGCTAGCAGCGAG	modify the nucleotide in *AtrpoA*
rpoA-CcAC-F	TATTACACGTGCAAAACCACAGAGCTAGCAGCGAG	modify the nucleotide in *AtrpoA*
rpoA-CcAA-F	TATTACACGTGCAAAACCAAAGAGCTAGCAGCGAG	modify the nucleotide in *AtrpoA*
KP1-seqF	CCACTCTGCTCGTGACAAAA	gene amplification and sequencing in editing analysis
KP2-seqF	GTCACCAAGAATCTGCGGAT	gene amplification and sequencing in editing analysis
KP3-seqF	GACTGCTGAACACACCCAGT	gene amplification and sequencing in editing analysis
KP4-seqF	ATCGCGTTTGGACTGATTTC	gene amplification and sequencing in editing analysis
KP5-seqF	CAGACCCTTCGAGTGACCAA	gene amplification and sequencing in editing analysis
KP6-seqF1	GGGCGGTGGGACGACGCATACCGGAT	gene amplification in editing analysis
KP6-seqF2	AATACGCCACAGGGACAGAC	sequencing in editing analysis
KP7-seqF	GACTCCTTAACACTCCTCAA	gene amplification and sequencing in editing analysis
T7short.REV	TATGCTAGTTATTGCTCAG	gene amplification and sequencing in editing analysis
PM18033-seqR	CACTGCATTCTAGTTGTGGTTTG	gene amplification and sequencing in editing analysis
