# Supplementary material for: U-to-C RNA editing by synthetic PPR-DYW proteins in bacteria and human culture cells
Source: Commun Biol. 2022 Sep 15;5:968. doi: 10.1038/s42003-022-03927-3 (PMC9478123; doi:10.1038/s42003-022-03927-3)
Supplement: Supplementary file 3 — Description of Additional Supplementary Files [file 42003_2022_3927_MOESM3_ESM.pdf]

Description of Additional Supplementary Files

File name: Supplementary Data 1
Description: Summary of RNA-seq analysis.

File name: Supplementary Data 2
Description: Potential off-target editing sites having one to three mismatches with AtrpoA editing site.

File name: Supplementary Data 3
Description: RNA editing analysis of DYW:KP protein variants in bacterial and human cells.

File name: Supplementary Data 4
Description: The source data behind the graphs depicting the editing efficiency of the KP proteins on AtrpoA in the paper.
