# Supplementary material for: U-to-C RNA editing by synthetic PPR-DYW proteins in bacteria and human culture cells
Source: Commun Biol. 2022 Sep 15;5:968. doi: 10.1038/s42003-022-03927-3 (PMC9478123; doi:10.1038/s42003-022-03927-3)
Supplement: Supplementary file 8 — Reporting summary [file 42003_2022_3927_MOESM8_ESM.pdf]

nature research

Corresponding author(s): Mizuho Ichinose and Bernard Gutmann

Last updated by author(s): Aug 31, 2022

Reporting Summary

Nature Research wishes to improve the reproducibility of the work that we publish. This form provides structure for consistency and transparency in reporting. For further information on Nature Research policies, see our Editorial Policies and the Editorial Policy Checklist.

Statistics

For all statistical analyses, confirm that the following items are present in the figure legend, table legend, main text, or Methods section.

n/a	Confirmed	
☐	☒	The exact sample size (*n*) for each experimental group/condition, given as a discrete number and unit of measurement
☐	☒	A statement on whether measurements were taken from distinct samples or whether the same sample was measured repeatedly
☐	☒	The statistical test(s) used AND whether they are one- or two-sided<br>*Only common tests should be described solely by name; describe more complex techniques in the Methods section.*
☒	☐	A description of all covariates tested
☒	☐	A description of any assumptions or corrections, such as tests of normality and adjustment for multiple comparisons
☐	☒	A full description of the statistical parameters including central tendency (e.g. means) or other basic estimates (e.g. regression coefficient) AND variation (e.g. standard deviation) or associated estimates of uncertainty (e.g. confidence intervals)
☐	☒	For null hypothesis testing, the test statistic (e.g. *F*, *t*, *r*) with confidence intervals, effect sizes, degrees of freedom and *P* value noted<br>*Give P values as exact values whenever suitable.*
☒	☐	For Bayesian analysis, information on the choice of priors and Markov chain Monte Carlo settings
☒	☐	For hierarchical and complex designs, identification of the appropriate level for tests and full reporting of outcomes
☒	☐	Estimates of effect sizes (e.g. Cohen's *d*, Pearson's *r*), indicating how they were calculated

Our web collection on statistics for biologists contains articles on many of the points above.

Software and code

Policy information about availability of computer code

Data collection	For direct sequencing, PCR products were purified (ExoSAP-IT Express PCR Cleanup Reagent, ThermoFisher) and sequenced directly (GENEWIZ).<br>For RNA-seq, RNA extraction was performed with Maxwell® RSC simplyRNA Tissue Kit (Promega) and Proteinase K. Libraries were prepared by GENEWIZ from total RNA using poly(A) enrichment of the mRNA
Data analysis	DYW domain sequences were aligned with MAFFT (v7.407)<br>Alignments were trimmed with TrimAl (v1.4.rev15)<br>FastTree (v2.1.10) was used for phylogenetic reconstitution.<br>EditR (http://baseeditr.com/) was used to analyze the sequencing chromatograms.<br>STAR (v2.9.7) was used to align fastq data<br>REDItools was used to determine the editing levels<br>Statistical analyses by the one-way ANOVA Tukey's comparison test was computed using R (v4.0.3)

For manuscripts utilizing custom algorithms or software that are central to the research but not yet described in published literature, software must be made available to editors and reviewers. We strongly encourage code deposition in a community repository (e.g. GitHub). See the Nature Research guidelines for submitting code & software for further information.

Data

Policy information about availability of data

All manuscripts must include a data availability statement. This statement should provide the following information, where applicable:

- Accession codes, unique identifiers, or web links for publicly available datasets
- A list of figures that have associated raw data
- A description of any restrictions on data availability

Uncropped and unedited images corresponding to the protein gels and blots are provided in Supplementary Figures 7 to 11. The data used to generate the graphs are available in Supplementary Data 1 and 3. Sanger sequence chromatograms used to score editing efficiencies are available as Supplementary Data 4. Transcriptome wide RNA-sequencing data are available at Sequence Read Archive under BioProject accession number PRJNA856069. Expression plasmids are available from addgene under a uniform biological material transfer agreement (accession IDs: 190955 to 190994). All other data are available from the corresponding authors on reasonable request.

Field-specific reporting

Please select the one below that is the best fit for your research. If you are not sure, read the appropriate sections before making your selection.

☒ Life sciences ☐ Behavioural & social sciences ☐ Ecological, evolutionary & environmental sciences

For a reference copy of the document with all sections, see nature.com/documents/nr-reporting-summary-flat.pdf

Life sciences study design

All studies must disclose on these points even when the disclosure is negative.

Sample size	Three biological replicates were used for each experiment.
Data exclusions	No data were excluded.
Replication	All attempts were successful
Randomization	This is not relevant to this study
Blinding	This is not relevant to this study

Reporting for specific materials, systems and methods

We require information from authors about some types of materials, experimental systems and methods used in many studies. Here, indicate whether each material, system or method listed is relevant to your study. If you are not sure if a list item applies to your research, read the appropriate section before selecting a response.

Materials & experimental systems

n/a	Involved in the study	
☐	☒	Antibodies
☐	☒	Eukaryotic cell lines
☒	☐	Palaeontology and archaeology
☒	☐	Animals and other organisms
☒	☐	Human research participants
☒	☐	Clinical data
☒	☐	Dual use research of concern

Methods

n/a	Involved in the study	
☒	☐	ChIP-seq
☒	☐	Flow cytometry
☒	☐	MRI-based neuroimaging

Antibodies

Antibodies used	Anti-His-tag mAb-HRP-DirectT, MBL D291-7
Validation	Validated by the manufacturer

Eukaryotic cell lines

Policy information about cell lines

Cell line source(s)	HEK293T (RIKEN, RCB2202)
Authentication	No authentication was performed
Mycoplasma contamination	The cell line was not tested for mycoplasma contamination
Commonly misidentified lines (See ICLAC register)	No commonly misidentified cell lines were used
